# Influence of Trace Elements on Stabilization of Aqueous Solutions of Ascorbic Acid

**DOI:** 10.1007/s12011-012-9524-4

**Published:** 2012-10-26

**Authors:** Barbara Dolińska, Aneta Ostróżka-Cieślik, Artur Caban, Klimas Rimantas, Lucyna Leszczyńska, Florian Ryszka

**Affiliations:** 1 Biochefa Pharmaceutical Research and Production Plant, Sosnowiec, Poland; 2 Department of Applied Pharmacy and Drug Technology, Silesian Medical Academy, Katowice, Poland; 3 Department of General, Vascular and Transplant Surgery, Medical University of Silesia Katowice, Katowice, Poland; 4 Department of Drug Technology, Kaunas University of Medicine, Kaunas, Lithuania

**Keywords:** Microelements, Vitamin C, Solutions, Preservation, Accelerated stability test

## Abstract

Together with vitamin C, zinc, selenium, manganese, and magnesium play a vital role in the preservation of organs scheduled for transplantation. In the present study, it is shown that addition of 1 mg/l of these elements influences the stability of 0.3 mM ascorbic acid solutions. The solution’s stability was estimated using an accelerated stability test. The concentration of vitamin C was measured using a validated spectrophotometric method, which uses the reduction of 2,6-dichlorophenoloindophenol by ascorbic acid. Elevated temperatures, the factor accelerating substances’ decomposition reaction rate, were used in the tests. The research was conducted at two temperatures at intervals of 10 °C: 80 ± 0.1 and 90 ± 0.1 °C. It was stated that the studied substances’ decomposition occurred in accordance with the equation for first-order reactions. The function of the logarithmic concentration (log%C) over time was revealed to be rectilinear. This dependence was used to determine the kinetics of decomposition reaction rate parameters. The stabilization of vitamin C solutions was measured as the time in which 10 % of the substance decomposed at 20 and 0 °C. Addition of Se(IV) or Mg(II) ions significantly increase the stability of ascorbic acid solution (∼34 and ∼16 %, respectively), but Zn(II) causes a significant decrease in stability by ∼23 %. Addition of Mn(II) has no significant influence on vitamin C stability.

## Introduction

Trace elements are essential for appropriate function of living organisms. Some elements can have a protective influence on selected organs during ischemia and reperfusion [1–6] and might have a positive role in stabilization of organ preservation solutions [7–9]. One such element is selenium, a component of glutathione peroxidase that plays a protective role against the oxidizing action of hydrogen peroxide [1–4].

Zinc influences cell membrane structure and function. It is a component of several enzymes and stimulates protein synthesis. It is a component of superoxide dismutase (Cu/Zn-SOD) and takes part in quenching of superoxide radicals. It was concluded that zinc is a HSP70 protein activator, which protects the liver during preservation [5].

Manganese is located at the active site of superoxide dismutase (Mn-SOD), which also eliminates superoxide anion radical. Increased activity of Mn-SOD is observed as a result of osmotic shock and action of reagents generating the anion radical [6]. Magnesium compounds are found in preservation solutions: ViaSpan, histidine–ketoglutarate–tryptophan (HTK), Celsior, Perfadex, Plegisol, and Polysol [7, 10, 11].

Preservation and perfusion solutions are enriched with anti-oxidizing agents, such as vitamin C. It helps protecting organs against ischemia–reperfusion damage, especially oxidative stress [10–13]. Vitamin C administration in rabbits during kidney transplantation reduces the effects of lipid peroxidation and improves performance of the graft [13]. Ascorbic acid used at 0.3 mM concentration is the most efficient antioxidant for HTK, Celsior, and ViaSpan solutions [14–17]. Solid vitamin C is relatively stable, but it decomposes rather quickly when dissolved in water. Factors such as pH, temperature, oxygen, and the presence of catalysts (iron, copper) influence the decomposition process. The lowest rate of oxidation is observed at pH 3, where vitamin C solutions are the most stable. Raising the pH to 5 increases the oxidation rate by a factor of 2. Preservation solutions need to mimic the physiological pH of serum (7.35–7.45), pH at which vitamin C is unstable.

For these reasons, we decided to study the stabilization effect of adding Zn(II), Se(IV), Mn(II), or Mg(II) at a concentration of 1 mg/l to 0.3 mM ascorbic acid, for use in preservation solutions for organs used in transplants. The time at which 10 % of the ascorbic acid is degraded at 20 °C was used as a measure of stabilization efficiency. Based on decomposition kinetics, a linear dependence between degradation speed and temperature was established.

## Materials and Methods

### Characteristics of Used Substances

l(+)-Ascorbic acid C_6_H_8_O_6_, mol. wt. = 176.13 g/mol; manganese sulfate [MnSO_4_·5H_2_O], mol. wt. = 241.07 g/mol; and zinc acetate [Zn(CH_3_COO)_2_], mol. wt. = 219.49 g/mol were purchased from POCh Gliwice, Poland. Sodium selenite [Na_2_SeO_3_], mol. wt. = 172.94 g/mol, was obtained from Sigma-Aldrich, Poland. Magnesium fumarate [C_4_H_2_MgO_4_], mol. wt. = 138.38 g/mol, was purchased from F.Z.N.P. “Biochefa.” All the reagents were analytically pure and met all necessary regulations.

### Solutions

Ascorbic acid stock solutions (0.3 mM) were produced using *pro injectione*-grade water. The selected stabilizing agents were added at 1 mg/l to the stock solution to produce the Zn(II), Se(IV), Mn(II), or Mg(II) study solutions. All solutions were prepared under Good Manufacturing Practice conditions in a laminar flow unit, filtered using a 0.22-μm Sartopure GF 2 cellulose acetate filter at a 50-ml/min flow rate. The solutions were stored in 10 ml hydrolytic class 1 glass vials, secured with rubber stoppers and aluminum caps.

### Measurement of the Vitamin C Concentration

The concentration of vitamin C was measured using a validated spectrophotometric method, which uses the reduction of 2,6-dichlorophenoloindophenol by ascorbic acid. A 1-mg/ml ascorbic acid solution in 2.5 % metaphosphoric acid was used as stock solution to construct a calibration curve in the 30 to 250 μg/ml range. The absorbance was read at 520 nm in 1.0-cm glass cuvettes using a CE 3021 UV–VIS Spectrophotometer (Cecil, England). The vitamin C concentration was calculated using an extinction coefficient *ε* = 5.17·10^4^·l·M^−1^ cm^−1^. The curve was fitted by the linear regression equation: *y* = −0.0244*x* + 0.6778 (*R* ^2^ = 0.9995; linear regression error, 0.00012).

### Solutions Stability Analysis Using Accelerated Stability Test

The accelerated stability test [14–18] was used to estimate the stability of vitamin C solutions based on the kinetics of the reaction rate of ascorbic acid concentration change at elevated temperatures. Vials were tested for tightness by immersing in 1 % methylene blue solution. Ten vials containing 0.3 mM ascorbic acid with or without the selected tested ions were placed in a water ultra thermostat (U7, temperature test precision ±0.1 °C). The test was run at two different temperatures: 80 °C (353 K) and 90 °C (363 K). These temperatures were chosen based on prior studies of ascorbic acid decomposition rate.

Samples were taken when temperature of the tested solution reached the desired temperature at 0 (100 % ascorbic acid), 1, 2, 3, 4, 6, and 8 h from the starting point of the experiment. Collected samples were cooled in water with ice directly after sampling in order to halt further decomposition of the substance. It was stated that the studied substances’ decomposition occurred in accordance with the equation for first-order reactions. The function of the logarithmic concentration (log%C) over time was revealed to be rectilinear. This dependence was used to determine the kinetics of decomposition reaction rate parameters. Based on the obtained data, the following were calculated: the decomposition reaction rate constants of ascorbic acid (*k*) at the temperature of 80 and 90 °C, temperature factor Q_10_, the decomposition rate constant of ascorbic acid at the temperature of 20 and 0 °C, and the stability at the temperature of 20 and 0 °C (*t*_10%_). The stability (*t*_10%_) is the time period after which ascorbic acid concentration decreases by 10 % in the solution. The decrease in ascorbic acid concentration by 10 % was calculated with the formula:

$$ t10\%=\frac{0.1053 }{{{k_{{(20\,^{\circ}\mathrm{C})}}}}}\,and\,10\%=\frac{0.1053 }{{{k_{{(0\,^{\circ}\mathrm{C})}}}}} $$

The decomposition rate constants of ascorbic acid (*k*) were determined at the temperature of 80 and 90 °C using the formula:

$$ k=\frac{{\ln \frac{C_0 }{{C_0 -{C_x}}}}}{t} $$

Where *C*_0_ is the concentration at *t* = 0 and *C*_*x*_ at any given time *t*.

The dependence of reaction rate on temperature is characterized temperature factor Q_10_:

$$ {Q_{10 }}=\frac{{k(90\,^{\circ}\mathrm{C})}}{{k(80\,^{\circ}\mathrm{C})}} $$

The solution stability at the temperature of 20 and 0 °C was calculated using the formula:

$$ {k_{{20\,^{\circ}\mathrm{C}}}}=\frac{{{k_{{80^{\circ}\mathrm{C}}}}}}{Q^6}\,and\,{k_{{0^{\circ}\mathrm{C}}}}=\frac{{{k_{{80^{\circ}\mathrm{C}}}}}}{Q^8 } $$

### Statistical Analysis

The results are presented as a mean value (*x*) and standard deviation calculated from 10 samples. The decomposition of ascorbic acid added with 1 mg/l Zn(II), Se(IV), Mn(II), or Mg(II) was compared to the decomposition of untreated control solution. The statistical significance was established at *p* < 0.05 using Student’s *t* test. Using variance analysis, we calculated the significance of microelements on ascorbic acid solution stabilization. All calculations were performed using Microsoft Excel and Statistica for Windows 5.1 software.

## Results

Preservation and perfusion solutions are a transient environment for organs used for transplantation. Consequently, the effectiveness and stability of the solution are crucial for safety of the transplant patients.

The degradation of aqueous solutions of ascorbic acid occurred at various rates depending on the trace element added. The slowest degradation was observed for Se(IV) and the fastest for Zn(II) ions. Table 1 shows the parameters describing the kinetics of ascorbic acid degradation in the study and control solutions. Analysis of variance has shown significant influence of added microelements on the stabilization of ascorbic acid solution (*F*(4,10) = 247,61; *p* < 0.0000).

**Table 1:** Parameters describing the kinetics of ascorbic acid degradation in the study and control solutions (means ± standard error)

Stability parameters	0.3 mM vit. C (control)	0.3 mM vit. C + Zn(II)	0.3 mM vit. C + Se(IV)	0.3 mM vit. C + Mn(II)	0.3 mM vit. C + Mg(II)
*C*_90°C_ (%)	51.14 ± 0.15	52.44 ± 0.73	60.43 ± 2.31*	51.14 ± 0.71	56.74 ± 1.82*
*C*_80°C_ (%)	76.44 ± 1.32	79.36 ± 1.43*	83.88 ± 1.52*	79.44 ± 1.11*	82.06 ± 1.15*
*k*_90°C_ (h^−1^)	0.082 ± 0.002	0.080 ± 0.002	0.062 ± 0.003	0.082 ± 0.001	0.070 ± 0.002
*k*_80°C_ (h^−1^)	0.035 ± 0.001	0.027 ± 0.005	0.021 ± 0.004	0.035 ± 0.003	0.024 ± 0.003
*t*_10 % 20 °C_(days)	94.98 ± 0.74	73.09 ± 1.07*	127.45 ± 1.92*	94.98 ± 0.93	110.44 ± 1.50*
*t*_10 % 0 °C_ (years)	2.20 ± 0.02	1.57 ± 0.04*	2.97 ± 0.07*	2.19 ± 0.03	2.56 ± 0.04*

*C*_*90 °C*_ *(%)* vit. C content after accelerated aging test at 90 °C, *C*_*80 °C*_ *(%)* vit. C content after accelerated aging test at 80 °C, *k*_*90 °C*_ *(h* ^*−1*^ *)* vit. C decomposition constant at 90 °C, *k*_*80 °C*_ *(h* ^*−1*^ *)* vit. C decomposition constant at 80 °C, *t*_*10 % 20 °C*_ *(days)* vit. C stability at 20 °C, *t*_*10 % 0 °C*_ *(years)* vit. C stability at 0 °C

*p < 0.05, statistically significant in comparison to controls—vit. C solution with no microelement added

As a result of the antioxidant properties of Se(IV), its addition to 0.3 mM ascorbic acid significantly increases the stability of the solution from ∼95 to ∼127 days, which is about 34 % over the stability of the control solution. Adding magnesium fumarate increased the stability of ascorbic acid solutions significantly, by ∼16 % to ∼110 days.

In the presence of Zn(II) ions, the stability of ascorbic acid significantly decreased by ∼23 % (∼73 days). This is probably a result of a catalytic action of Zn(II) ions on ascorbate oxidation. Addition of Mn(II) had no significant effects on the stability of ascorbic acid.

## Discussion

Preservation and perfusion solutions must be water-based. Free protons and hydroxide anions that might be present in aqueous solutions have the potential to generate free radicals. As a strong polar solvent, water is an ideal environment favoring decomposition reactions that decrease solution stability and effectiveness. Addition of suitable antioxidants is therefore crucial for maintaining the stability of solutions that will be used in preservation of transplant organs.

Adding Se(IV) to 0.3 mM ascorbic acid solution significantly increases its stability. Additionally, selenium ions can play a vital role in minimizing ischemia–reperfusion damage in kidneys during perfusion, preservation, and reperfusion. It was concluded that addition of selenium to preservation solutions protects kidneys against oxidative stress during ischemia [1]. A decrease in the concentration of malondialdehyde was seen 2 h after kidney transplantation as a result of adding selenium to HTK solution [2]. Selenium addition to Euro-Collins (EC) solution proved higher efficiency in lung protection during ischemia–reperfusion than the original EC solution [3]. Addition of sodium selenite to a preservation solution at an optimal concentration of 0.5 μM positively influences heart function after warm ischemia [4].

Magnesium ions also cause increase in vitamin C stability. It was also proved that magnesium ions stabilize cell membranes and activate enzymes responsible for synthesis, storage, and assimilation of high-energy compounds. Magnesium ions Mg(II) were added in the form of fumarate because fumarate shows positive influence in cardioplegia solutions. It helps myocardial contractility because it causes a decrease of lactic dehydrogenase, which increases with the number of dead cells [19].

Ascorbate can act as a monodentate ligand that reacts with metal cations in aqueous solutions resulting in low stability water-soluble complexes. This complexation reaction involves the lactone ring and the side chain [20]. By decreasing the amount of free cations, precipitation does not occur, increasing the stability of the solutions.

Addition of Se(IV) and Mg(II) ions to 0.3 mM ascorbic acid solutions significantly increases its stability but Zn(II) decreases it. The addition of Mn(II) has no significant influence on the stability of ascorbic acid.
